# Occupational reintegration after severe burn injury: a questionnaire study

**DOI:** 10.1007/s00508-021-01871-6

**Published:** 2021-04-28

**Authors:** Vera Vorstandlechner, Daniel Langthaler, Katharina Ebenberger, Anna Pittermann, Gerald Ihra, Thomas Rath, Jakob Nedomansky, Gabriela Muschitz, Christine Radtke, Alexandra Fochtmann-Frana

**Affiliations:** 1 Clinical Division of Plastic and Reconstructive Surgery, Department of Surgery, Medical University of Vienna, Waehringer Guertel 18–20, 1090 Vienna, Austria; 2 Clinical Division of Plastic and Reconstructive Surgery, Department of Surgery, Department of Clinical Psychology and Psychotherapy, Medical University of Vienna, Vienna, Austria; 3 Department of Anesthesiology and General Intensive Care, Medical University of Vienna, Vienna, Austria

**Keywords:** Burns, Wounds and injuries, Surveys and questionnaires, Occupations, Work after burn injury

## Abstract

**Background:**

As a consequence of improved survival rates after burn injury occupational reintegration of burn survivors has gained increasing significance. We aimed to develop a precise patient questionnaire as a tool to evaluate factors contributing to occupational reintegration.

**Material and methods:**

A questionnaire comprising 20 questions specifically evaluating occupational reintegration was developed under psychological supervision. The single-center questionnaire study was implemented in patients with burn injuries who were admitted to the 6‑bed burn intensive care unit (BICU) of the General Hospital of Vienna, Austria (2004–2013). The questionnaire was sent to burn survivors of working age (18–60 years) with an abbreviated burn severity index (ABSI) of 6 or greater, a total burn surface area (TBSA) of 15% or greater, and a BICU stay of at least 24 h.

**Results:**

A total of 112 burn survivors met the inclusion criteria and were contacted by mail. Of the 112 patients 11 (10%) decided to participate in the study and 218/220 questions (99%) in 11 patients were answered. Out of 11 patients 7 (64%) reported successful return to work and 4 of 11 (36%) did not resume their occupation. Advanced age, longer BICU and hospital stays, higher TBSA, burn at work, lower education, and problems with esthetic appearance seemed to impair patients’ return to their occupation.

**Conclusion:**

When implementing the questionnaire, severely burned patients with higher age, lower education, and longer hospital and BICU stay seemed at high risk for failed reintegration in their profession after burn injury.

## Introduction

Because of enhanced surgical treatment and intensive care medicine, the survival of burn patients was significantly increased during the last decades. Consequently, the need for full social and economic reintegration after the burn injury has increased [1]. Return to employment after a burn injury can be considered to be the best functional outcome possible after surviving a severe burn [2]. Pain, scarring, and adaption to the post-burn esthetic appearance are difficulties that need to be overcome to achieve full socioeconomic rehabilitation after a burn injury [3, 4]. Previous studies showed the great importance of return to employment for the patient’s health and well-being. Along with an income, satisfaction of psychosocial needs and one’s individual identity are based on a person’s occupation [2, 5, 6]. Failure of return to work resulted in lower health-related quality of life [7]. Moreover, lack of socioeconomic reintegration and absenteeism from work after burn injury contribute to the already high costs of a burn injury [8].

Factors described previously as possibly impairing a patient’s return to work were greater extent of the burn injury, burn injury of the hands, burn injury as an occupational accident, longer hospital stay, and higher age [2, 4, 5, 9]. Furthermore, the presence of comorbidities, pain, inhalational injury and lower educational status seem to predict the socioeconomic and occupational outcome of patients after severe burn injury [4, 10, 11].

The rationale of this study was to specifically address the psychological issues and occupational recovery and reintegration of patients after burn injury. Therefore, no standardized questionnaire (e.g. the 36-item short-form survey, SF36, a nonspecific questionnaire to assess a patient’s life quality) was applicable. We aimed to gain precise data on burn patients’ return to employment after burn injury and their occupational situation after treatment in a burn center.

## Patients, material and methods

A questionnaire of 20 questions was designed under psychological supervision. The patients were asked about their professional education, highest level of education, career, occupational situation before and after the burn injury, duration of employment before burn injury, mode of employment, dismissal after burn injury and reintegration into their profession. In addition, questions concerning duration of rehabilitation, start of occupation after rehabilitation, or dismissal from work during rehabilitation were answered. The patients were asked to answer questions concerning difficulties with their esthetic appearance and difficulties during their occupational reintegration process. The questions were designed as either closed question format (yes/no), multiple or single choice.

A retrospective questionnaire study of all patients admitted to the Vienna General Hospital burn intensive care unit (BICU) between January 2004 and December 2013 was performed. The inclusion criteria were defined as follows: age between 18 and 60 years, abbreviated burn severity index (ABSI) of at least 6, a total burn surface area (TBSA) of at least 15% and a BICU stay of at least 24 h. Patients had to have been discharged from the rehabilitation clinic for at least 12 months. These patients were contacted via mail with an invitational letter explaining the study, an informed consent form, the questionnaire concerning their occupational situation and a post-paid return envelope. Clinical data were recorded and analyzed according to age, gender, mode of injury, TBSA, ABSI, inhalation trauma, surgical procedures, and duration of BICU and hospital stays. Approval from the local ethics committee (EC No. 1078/2014) was obtained.

Statistical analysis was performed using Microsoft Excel 2016 (Microsoft, Redmond, USA) and GraphPad Prism 5 (GraphPad Software, LaJolla, USA). Scale variables are described using mean, minimum, maximum, and standard variation, and are displayed in column graphs. Column analysis was performed using means and standard variation. An unpaired t test was used to compare the means of the groups (95% confidence interval, CI). Patients who answered the study invitation (answer, *n* = 11) were compared to those who did not (no answer, *n* = 81). Scale variables were analyzed using frequency distribution and cross tables.

## Results

**Fig. 1:**
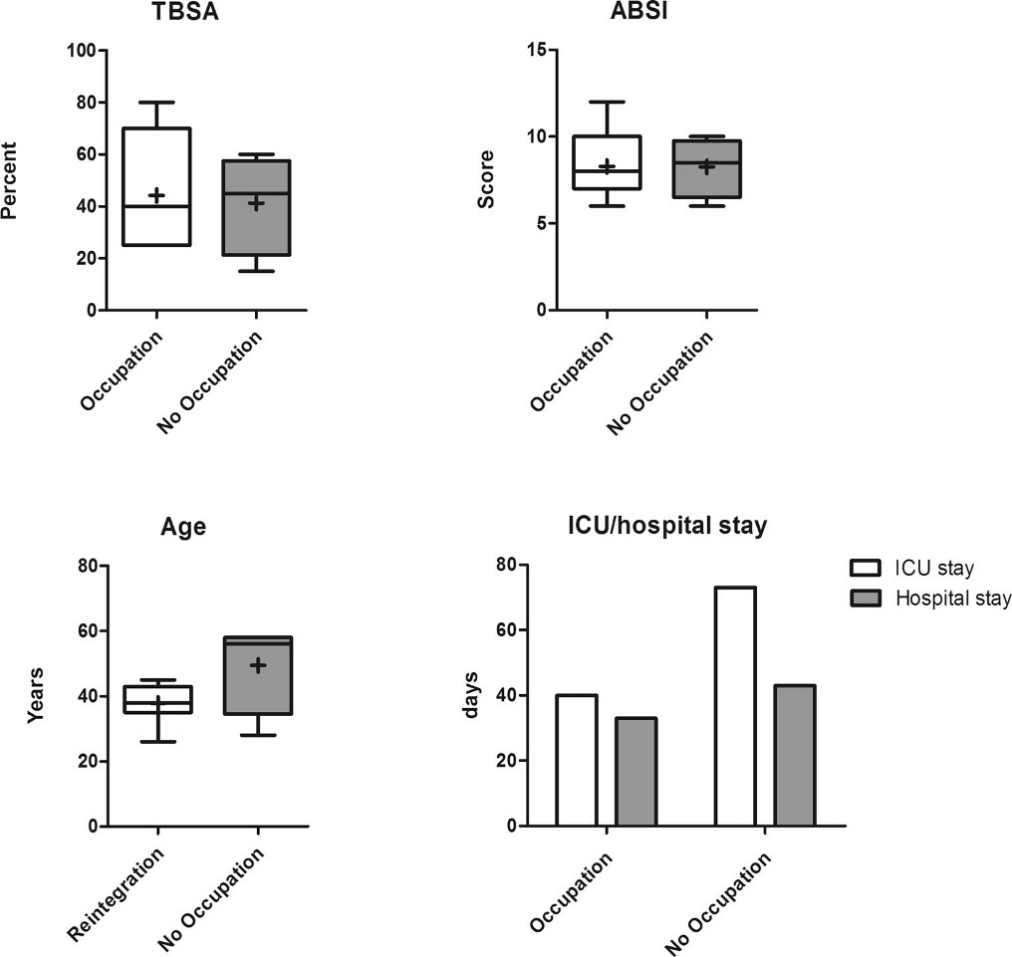
Demographic data*.* Boxplot and column graphs show the patients’ medical and demographic data. Patients who returned to an occupation after burn injury (occupation, *n* = 7) are compared with those who did not (no occupation, *n* = 4). Total burn surface area (TBSA), Abbreviated Burn Severity Index (ABSI), and age are given in boxplot diagrams. *Line* represents median, *+* represents mean, *boxes* represent lower and upper quartile and *whiskers* represent minimum to maximum. Columns of hospital stay represent days in hospital, comparing occupation and no occupation

A total of 112 patients were contacted for study participation and 11 (10%) patients decided to participate: 7 of 11 (64%) of the study participants were successfully reintegrated into an occupation (group occupation), 10 of 11 (91%) of these patients were male and 1 patient was female. Four of 11 (36%) of the participants reported no occupation after their burn injury (no occupation group), all of whom were male (Fig. 1). Patients who reintegrated into an occupation (occupation) were younger than those in the no occupation group (38 years, range 26–45 years vs. 50 years, range 28–58 years, *p* = 0.09). The mean TBSA in the occupation group was 44% (range, 25–80%) versus 41% (range, 15–60%) in the no occupation group (*p* = 0.82). The mean ABSI in the occupation group was 8 (range, 6–12) versus 8 (range, 6–10) in the no occupation group (*p* = 0.97). All patients included in the current study had deep dermal or full thickness burn injuries. Only one patient had an inhalation trauma; he reported having an occupation after his burn injury. The average overall hospital stay of patients returning to an occupation was 40 days (range, 12–92 days); those patients who did not return to work stayed an average of 74 days (range, 21–103 days) (*p* = 0.13) (Fig. 1). The mean BICU stay was 34 days (range, 7–65 days) in the occupation group versus 44 days (range,1–89 days) in the no occupation group (*p* = 0.59).

Out of 11 individuals 2 (18%) in the occupation group and 2 out of 4 (50%) in the no occupation group had facial and/or hand burns. In two of seven (42%) patients in the occupation group and three of four (75%) in the no occupation group, the burn injury was caused by an occupational accident (*p* = 0.24). Out of seven patients who returned to an occupation six (85%) reported that they were satisfied with their current profession. All patients in the occupation group returned to their field of profession from before the burn injury. Three of seven (42%) changed to another company/employer, and one patient was dismissed from the previous job after the burn injury.

Seven of 11 (63%) individuals reported apprenticeship as the highest degree of education; 1 of 11 (10%) reported high school, and 3 of 11 (27%) had an academic degree. Three of 7 (43%) patients in the occupation group held an academic degree, and 4 of 7 (57%) had a graduation of apprenticeship (*p* = 0.23). In the occupation group patients stayed an average of 8 weeks (range, 0–36 weeks) in the rehabilitation hospital. The no occupation patients stayed in the rehabilitation facility an average of 13.5 weeks (range, 0–26 weeks) (*p* = 0.53). Three of seven (43%) patients returned to their job less than 1 month after discharge from the rehabilitation hospital. None of the no occupation patients are currently planning on returning to an occupation. Two of them went into early retirement, and two went into regular retirement.

Four of 11 (36%) patients reported medical problems (1/7, 14% in occupation group, ¾, 75% in no occupation group). Among these, pain (*n* = 3), reduced mobility (*n* = 3), neurological problems (*n* = 1), psychological problems (*n* = 1), and other problems (*n* = 1) were mentioned in the questionnaire. One occupation group patient had pruritus. Five of 11 (45%) patients (2/7, 29% in occupation group, ¾, 75% in no occupation group) patients stated problems with their esthetic appearance.

Regarding patient demographics, we compared patients who answered the study invitation (answer, *n* = 11) with individuals who decided not to answer the study invitation (no answer, *n* = 81). The mean TBSA was slightly greater in the group of patients who decided to answer the questionnaire compared with individuals who decided not to answer the questionnaire (43%, range 15–80% vs. 37%, range 15–80%, *p* = 0.29). The mean ABSI was the same in both groups (8, range 6–12 vs. 8, range, 6–14, *p* = 0.58). The mean age did not differ between groups (42 years, range 26–58 years in answer group vs. 41 years, range 18–60 years in no answer group, *p* = 0.79). The mean hospital stay among patients who answered the questionnaire compared with individuals who did not answer differed to a statistically significant degree (43 days, range 12–103 days vs. 54 days, range 7–269 days, *p* = 0.91). The BICU stay did not show any statistical difference between the groups (37 days, range 1–89 days in answer group vs. 42 days, range 2–265 days in no answer group, *p* = 0.68).

## Discussion

We used a new questionnaire to investigate characteristics of occupational reintegration in patients after severe burn injury. We provide precise data on patients with large burns and their course of professional or occupational reintegration. Furthermore, we were able to analyze burn-related data of 112 patients in a study period of 10 years; however, the study is limited by the small sample size of patients who replied to the questionnaire. With only 10% of patients replying to the study invitation, this raises the question of the causes of the small response. On the part of our ethics committee, we were not allowed to contact the former patients by telephone. We were only allowed to contact them by mail. This fact may explain the low number of responses. We hypothesize that it is due to the psychological difficulties that accompany surviving a severe burn injury. Memories or associations with their injury and hospital treatment are probably a strong stressor for the patients and therefore avoided as severe burn injuries are often associated with posttraumatic anxiety and depression [12]. A systematic review on occupational reintegration demonstrated a cross-study number with 72% of patients returning to any form of occupation [3]. A prospective study found 67% of participants resuming their work [5]. Although our sample size was small these data of the percentage of patients who were able to work after their injury are in accord with our numbers of 7/11 (64%).

In addition to that it was previously suggested that a higher TBSA and ABSI, full-thickness burns, mean time off work and duration of treatment as factors mainly influencing successful return to an occupation [8, 11, 13–15]. As TBSA and ABSI did not differ between groups, these were not found as predictors for reintegration in our study. Nevertheless, in the present study we found a strong tendency of higher age and longer hospital or BICU stay to possibly impair successful integration. Higher age was reported as a predictor for failure of return to work by others [15–17]; however, in contrast to the data of the present study, age was found to be a poor predictor in a systematic review [3]. Nevertheless, based on the present study we assume that higher age is an important risk factor impairing the patients’ chances for return to their occupation.

Longer ICU and hospital stays were found before as major factors contributing to the duration until return to work or successful occupational reintegration [2, 5, 14]. The results of the present study are broadly consistent with these previous findings. The average hospital stay of the no occupation group was 85% longer than those in the occupation group in the present collective. Considering the duration of inpatient rehabilitation stay, patients who did not return to work had an average 62% longer stay than those who did not. Elongated time until successful occupational reintegration was also described in patients who underwent inpatient rehabilitation, indicating that rehabilitation correlates with return to work [2].

Another predictor for successful return to work that was discussed previously by other authors is patient education level at the time of injury [17]. We found this aspect to be also contributing to the burn outcome: all patients who held an academic degree at the time of injury were able to resume their profession afterward. Related to this, the circumstance of the accident noted as burn injury at work seems to contribute to failure of reintegration in the present study and has similarly been reported by others as an important factor of failure of occupational reintegration [2].

We suspect that older patients with lower educational level are at the highest risk for failure of occupational reintegration. The factors mentioned previously should be considered during the hospital stay, and afterward during the rehabilitation stay. An implication of these findings is that burn patients who suffered from a burn injury that occurred as an occupational accident, older patients and patients with a low level of education should always undergo intensive psychological care as well as occupational therapy and physiotherapy.
